# Socioeconomic determinants of cumulative fertility in Ghana

**DOI:** 10.1371/journal.pone.0252519

**Published:** 2021-06-01

**Authors:** Samuel H. Nyarko

**Affiliations:** 1 Department of Demography, College for Health, Community & Policy, The University of Texas at San Antonio, San Antonio, United States of America; 2 Department of Population and Behavioural Science, School of Public Health, University of Health and Allied Sciences, Hohoe, Ghana; University of Salamanca, SPAIN

## Abstract

The pace of decline in fertility rates in sub-Saharan Africa appears to have slowed or stalled in the last few decades. This study examines the socioeconomic associated with cumulative fertility in Ghana. Negative binomial regression models were used to estimate determinants of cumulative fertility using data from the Ghana Demographic and Health Surveys of 2003, 2008, and 2014. The composition of children ever born by women did not change considerably over the study periods. Socioeconomic disparities in educational attainment, household wealth, employment, and employer status are significantly associated with cumulative fertility risks in Ghana. The current age of women, age at sexual debut, and marital status, among others, are also linked to cumulative fertility levels. Place and region of residence are also linked to fertility in Ghana. Cumulative fertility levels in Ghana are underscored by considerable socioeconomic disparities among women of reproductive age. Fertility regulation policies should hinge on improving the socio-economic wellbeing of women in Ghana.

## Introduction

Fertility is one of the three main components of population dynamics that shape the size, structure, and composition of the human population. For the past few decades, world fertility decline has gained momentum, reaching historically low rates between 2010 and 2015 [1], and this trend is expected to continue until only a few countries have high fertility rates [2]. Despite the fertility decline, recent evidence shows that ongoing fertility declines have slowed or stalled in many countries in transition in sub-Saharan Africa [3,4]. As a corollary, fertility remained at relatively high rates (more than 3.5 births per woman) between 2010 and 2015 in about 42 countries in Africa [1].

Similarly, in Ghana, fertility has declined slowly from 6.4 children per woman in 1988 to 4.2 children per woman in 2014 [5], a drop of just about two births per woman over twenty-six years, while evidence from the 2017 Ghana Maternal Health Survey (GMHS) shows that total fertility has reduced to 3.9 [6]. Meanwhile, the 1994 National Population Policy of Ghana sought to reduce the total fertility rate from 5.5 to 3.0 by 2020 [7]. Per the rate of decline, it is not surprising that the total fertility rate target of 3.0 per woman stipulated by the National Population Policy was not attained by 2020. High fertility is found to be caused by a lower level of social and economic development, weaker family planning programs, high unmet need for family planning, higher ideal family size, and high adolescent fertility rates in sub-Saharan African countries [3,8]. Ghana’s family planning indicators do not appear encouraging either, as only about a quarter of all women ages 15–49 use contraceptives [6], with over one-third facing an unmet need for family planning [9]. It is against this backdrop that The Ghana Family Planning Costed Implementation Plan (2016–2020) was implemented as part of the global Family Planning 2020 (FP2020) agenda to considerably improve the demand for family planning services by removing user fees for family planning services in public health facilities [10].

The potential effects of higher fertility on the human population may be diverse. Fertility combined with mortality and migration affects the structure of the human population and has negative implications for child and maternal health, child schooling, the demographic dividend as well as the natural environment [11]. The slower pace of fertility declines, together with the high fertility rates means that in the absence of policies seeking to accelerate fertility decline, sub-Saharan African countries will continue to experience rapid population growth that in turn will constrain their development [12]. Meanwhile, studies on fertility determinants in Ghana have mainly focused on a comparative analysis between Ghana other sub-Saharan African countries such as Ivory Coast [13], Nigeria [14,15], and Kenya [16]. Besides, scores of studies in Ghana have examined various determinants of mainly fertility in the previous five years instead of lifetime fertility [17–20]. There is, however, a dearth of knowledge on the determinants of lifetime fertility levels among women of childbearing age in Ghana. To fill this gap, therefore, this study mainly examines the socioeconomic factors associated with cumulative fertility in Ghana using pooled nationally representative data. The study provides a better understanding of the linkage between socioeconomic inequalities as well as demographic characteristics and cumulative fertility levels in Ghana and, in turn, offers appropriate policy directions for fertility regulation programs in the country.

## Data and methods

### Data source

The data source for this research is the 2003, 2008, and 2014 waves of the Ghana Demographic and Health Surveys (GDHS). These are three consecutive surveys conducted by the Ghana Statistical Service in collaboration with the Noguchi Memorial Institute for Medical Research (NMIMR), Ghana Health Service, and ICF Macro, among others [5], as part of the worldwide series of the United States Agency for International Development (USAID)-funded demographic and health surveys program. The GDHS is a nationally representative survey that provides data on the full birth history of women in their reproductive age (15–49 years); consequently, it serves as the germane source of data for this study. The 2003 GDHS comprises 5,691 women ages 15 to 49 and 5,015 men ages 15 to 59 selected from 6,251 households covering 412 clusters across the country [21] while the 2008 GDHS comprised 4,916 women and 4,568 men from 6,141 households covering 412 clusters [22]. The 2014 GDHS also includes 9,396 women and 4,388 men selected from 11,835 households that covered 427 clusters nationwide [5]. The surveys used a two-stage sample design based on the sampling frame of the 2000 and 2010 decennial Ghana Population and Housing Censuses. The first stage involved the selection of clusters while the second stage involved the systematic sampling of households [5,21,22]. Even though the 2017 GMHS has more current information, it was not used because this study is aimed at leveraging pooled data from three consecutive and more regular surveys to allow for the assessment of time trends in fertility risks, rather than focusing on a single decennial survey (GMHS 2017). The GDHS is a secondary (third party) open data source and, as such, an ethics statement is not applicable. The unit of analysis is all women ages 15 to 49 irrespective of their marital status and number of children ever born. A total sample size of 20,003 women was obtained from the three waves for the analysis.

### Study variables and measurements

Cumulative fertility (number of children ever born) of women, which is a count outcome, was used as the outcome variable for this study. The main independent variables considered in this study included socioeconomic characteristics such as education (no education, primary, secondary/higher), wealth status (poor, middle, rich), work status (working, not working), employment period (all year, seasonal) and employer status (Self-employed, someone else). These variables comprise the main interest of the current study by serving as the foundation of the analysis.

The control variables–potential confounding factors–considered in this analysis included demographic characteristics such as age group (<20, 20–29, 30–39, 40+), age at first sex (<20, 20–29, 30+), marital status (never married, married/cohabiting, separated/divorced/widow), ethnicity (Akan, Ga/Dangme, Ewe, Mole-Dagbani, Others), and religion (measured as Christianity, Islam, Traditional/Spiritual, Others). Spatio-temporal factors such as place of residence (rural, urban), region of residence as well as survey year (2003, 2008, 2014) were as well considered for the control variables. The inclusion of these variables in the current study was mainly informed by the significant associations observed between these variables and fertility by several previous studies [23–25].

### Analytic strategy

The data processing and analysis were done with the R statistical software (version 3.5.1) [26]. Descriptive results were initially provided to describe the background characteristics of the respondents and fertility in the sample. Furthermore, negative binomial regression models were estimated to examine the determinants of cumulated fertility and 95 percent confidence intervals.

The negative binomial regression model was used because it includes a dispersion parameter and better accounts for the observed overdispersion in the outcome variable (See supplementary file) than the Poisson regression model [27]. This was modeled as follows: log (Children_*i*_) = *β*_*0*_ *+ β*_*1*_*X*_*1*_ *+ β*_*2*_*X*_*2+*_ *β*_*3*_*X*_*3*_; where the log of children born to a woman ‘*i*’ is equal to the intercept (β_0_) plus the predictor variables (*β*_*1*_*X*_*1*_ *+ β*_*2*_*X*_*2+*_ *β*_*3*_*X*_*3*_) to be estimated. Three negative binomial regression models were fitted to estimate the population parameters, such as the betas and confidence intervals.

Model 1 was used to examine risk levels of only socioeconomic characteristics before adjusting for control variables. Model 2 estimated the significance of the socioeconomic characteristics after adjusting for the demographic characteristics of respondents. Model 3, which is the full model, examined the significance of the socioeconomic factors after adjusting for both demographic characteristics and spatio-temporal factors such as place and region of residence and the survey year. The estimated population parameters were used to calculate risk ratios and 95 percent confidence intervals for the results. The risk ratios show how much the expected number of children is multiplied compared to the reference group. Owing to their pooled nature, the data were de-normalized by multiplying the standard weight by the ratio of estimated total females ages 15–49 in the country in 2003, 2008, and 2014 [28] to the number of women interviewed in the respective surveys [29]. The de-normalized data were then weighted using the complex survey design to adjust for the multi-stage sampling nature of the data and to produce accurate and nationally representative results.

## Results

### Descriptive results

This section of the paper describes the sociodemographic characteristics of the respondents as presented in Table 1 below. Over one-third of the respondents were women ages 20 to 29(34.4%) while about one-fifth (19.0%) were women ages 15–19. About 59 percent of the respondents had secondary school or higher education while more than one-fifth (22.2%) had no formal education. The respondents were predominantly Christians (84.2%) while Muslims comprised over one-tenth (11.7%). The Akans (52.8%) made up more than half of the respondents while the Ga-Dangmes made up the least (7.7%).

**Table 1:** Percentage of the sample by background characteristics.

Characteristics	2003	2008	2014	2003–2014
**Age group**	**%(N = 5,691)**	**%(N = 4,916)**	**%(N = 9,396)**	**%(N = 20,003)**
15–19	20.2(1,150)	20.8(1,022)	17.3(1,626)	19.0 (3,801)
20–29	34.5(1,963)	34.8(1,711)	34.2(3,213)	34.4(6,881)
30–39	26.8(1,525)	26.1(1,283)	28.4(2,668)	27.4(5,481)
40–49	18.5(1,053)	18.3(900)	20.1(1,889)	19.2(3,840)
**Education level**				
No education	28.2(1,605)	21.2(1,042)	19.1(1,795)	22.2(4,440)
Primary education	20.0(1,138)	20.1(988)	17.8(1672)	19.0(3,801)
Secondary/higher	51.8(2,948)	58.7(2,886)	63.1(5,929)	58.8(11,762)
**Religious affiliation**				
Christianity	97.1(5,526)	77.5(3,810)	80.1(7,526)	84.2(16,843)
Islam	2.8(159)	15.0(737)	15.2(1,428)	11.7(2,340)
Traditional/Spiritual /Other	0.1(6)	7.5(369)	4.7(442)	4.1(820)
**Ethnicity**				
Akan	53.2(3,028)	53.2(2,615)	52.4(4,924)	52.8(10,562)
Ga-Dangme	8.2(467)	7.0(344)	7.7(723)	7.7(1,540)
Ewe	13.1(745)	12.9(634)	13.5(1,268)	13.2(2,641)
Mole-Dagbani	12.8(728)	16.1(792)	14.8(1,391)	14.6(2,920)
Other	12.7(723)	10.8(531)	11.6(1,090)	11.7(2,340)
**Wealth status**				
Poor	33.7(1,918)	34.3(1,686)	33.5(3,148)	33.8(6,761)
Middle	18.8(1,070)	19.9(978)	20.6(1,935)	19.9(3,981)
Rich	47.5(2,703)	45.8(2,252)	45.9(4,313)	46.3(9,261)
**Marital status**				
Never married	28.4(1,616)	32.4(1,593)	32.9(3,091)	31.5(6,301)
Married/living together	62.4(3,551)	58.5(2,876)	56.6(5,318)	58.7(11,742)
Widow/divorced/separated	9.2(524)	9.1(447)	10.5(987)	9.8(1,960)
**Work status**				
Working	75.2(4,280)	75.3(3,702)	73.5(6,906)	74.4(14,882)
Not working	24.8(1,411)	24.7(1,214)	26.5(2,490)	25.6(5,121)
**Residence**				
Urban	48.4(2,754)	48.5(2,384)	53.8(5,055)	50.9(10,182)
Rural	51.6(2,937)	51.5(2,532)	46.2(4,341)	49.1(9,821)
**Region**				
Western	9.7(552)	9.1(447)	11.0(1,034)	10.2(2,041)
Central	7.6(433)	8.6(423)	10.0(940)	9.0(1,800)
Greater Accra	16.5(939)	17.3(850)	20.2(1,898)	18.5(3,701)
Volta	8.6(489)	8.8(432)	7.7(723)	8.2(1,640)
Eastern	10.6(603)	9.8(482)	9.3(874)	9.8(1,960)
Ashanti	20.0(1,138)	20.6(1,013)	19.1(1,795)	19.7(3,941)
Brong Ahafo	10.0(569)	8.7(428)	8.2(770)	8.8(1,760)
Northern	8.8(501)	9.5(467)	8.4(789)	8.8(1,760)
Upper East	2.7(154)	5.1(251)	3.8(357)	3.8(760)
Upper West	5.5(313)	2.5(123)	2.3(216)	3.2(640)

Source: GDHS 2003–2014.

Respondents from rich households comprised 46.3 percent of the total sample while respondents from poor households comprised about one-third (33.8%). Further, about 59 percent of the respondents were married or living together as partners while 31.5 percent were never married or living together with any partner. Also, the majority of the respondents were in the labor force (74.4%) whereas a quarter was not working (25.6%). Regarding residence, about half of the respondents comprised those from urban settings (50.9%). The Ashanti (19.7%) and Greater Accra (18.5%) Regions had the highest representation of the total study sample while the Upper West Region (3.2%) had the least representation.

**Fig 1:**
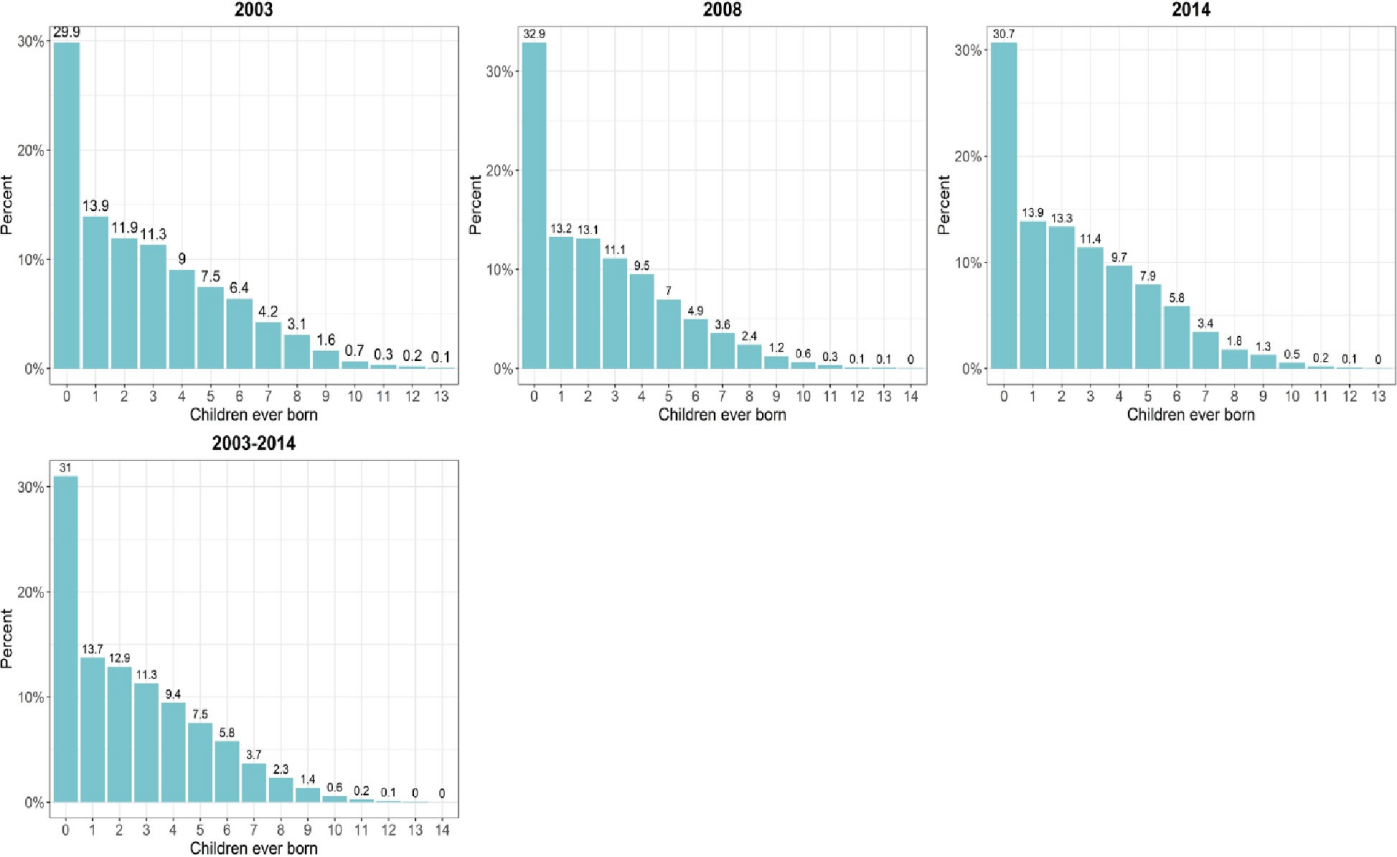
Cumulative fertility of women in the sample.

Fig 1 shows the descriptive results on the individual numbers of children ever born by women in the sample for each survey year for the study period. The average percentage of women who were nulliparous (never had children) for the study period was 31.0 percent while about 42 percent of the women in the sample had 3 or more children. The parity composition of the study sample appeared to have fluctuated marginally by survey year over the study period and did not show any considerable reduction over time from 2003 to 2014.

### Multivariate analysis results

Table 2 presents a summary of the results of three negative binomial regression models fitted to examine the socioeconomic characteristics affecting cumulative fertility. The results from the Wald test show that models 2 (379.88, p = 0.000) and 3 (5.53, p = 0.000) fit the data better than model 1. In model 1, the results show that all but one of the socioeconomic characteristics had a significant association with cumulative fertility when demographic and residential characteristics were not controlled. Even after controlling for demographic and residential factors in models 2 and 3, respectively, the significance of three of these socioeconomic variables persisted although their strength had waned slightly, whereas the significance of one had attenuated considerably.

**Table 2:** Negative binomial regression analysis of cumulative fertility in Ghana.

Variables	Model 1	Model 2	Model 3
	RR (95%CI)	RR (95%CI)	RR (95%CI)
**Education level** (Ref = No education)			
Primary education	0.78(0.75, 0.81)*	0.94(0.90, 0.97)*	0.94(0.91, 0.98)*
Secondary/higher	0.59(0.57, 0.62)*	0.79(0.76, 0.82)*	0.80(0.77, 0.83)*
**Wealth status** (Ref = Poor)			
Middle	0.92(0.88, 0.96)*	0.92(0.88, 0.95)*	0.93(0.89, 0.96)*
Rich	0.71(0.68, 0.74)*	0.73(0.70, 0.75)*	0.76(0.73, 0.80)*
**Work status** (Ref = Working)			
Not working	0.70(0.63, 0.79)*	0.93(0.85, 1.01)	0.93(0.85, 1.01)
**Employer status** (Ref = Someone else)			
Self-employed	1.91(1.82, 2.00)*	1.12(1.08, 1.16)*	1.11(1.07, 1.15)*
**Employment period** (Ref = All year)			
Seasonal	0.92(0.89, 0.96)*	1.01(0.98, 1.05)	1.00(0.97, 1.04)
**Age group** (Ref = 15–19)			
20–29		2.66(2.31, 3.05)*	2.67(2.32, 3.07)*
30–39		5.25(4.57, 6.04)*	5.31(4.61, 6.11)*
40–49		7.30(6.34, 8.42)*	7.38(6.39, 8.52)*
**Age at first sex** (Ref = <20)			
20–29		0.77(0.75, 0.80)*	0.78(0.75, 0.80)*
30+		0.25(0.15, 0.40)*	0.25(0.15, 0.39)*
**Marital status** (Ref = Never married)			
Married/living together		3.81(3.44, 4.21)*	3.76(3.39, 4.16)*
Widowed/Sep/Divorced		3.20(2.88, 3.55)*	3.18(2.86, 3.53)*
**Religious affiliation** (Ref = Christianity)			
Islam		1.02(0.97, 1.07)	1.03(0.98, 1.08)
Traditional/Spiritual		1.10(1.04, 1.17)*	1.12(1.06, 1.20)*
Other		1.02(0.95, 1.10)	1.05(0.97, 1.13)
**Ethnicity** (Ref = Akan)			
Ga-Dangme		0.91(0.87, 0.96)*	0.98(0.92, 1.03)
Ewe		0.89(0.86, 0.93)*	0.95(0.90, 1.00)
Mole-Dagbani		0.90(0.86, 0.95)*	0.95(0.90, 1.01)
Other		0.92(0.88, 0.97)*	0.96(0.91, 1.01)
**Year (Ref = 2003)**			
2008			0.96(0.93, 0.99)*
2014			0.94(0.92, 0.97)*
**Place of residence** (Ref = Urban)			
Rural			1.05(1.01, 1.09)*
**Region** (Ref = Greater Accra)			
Western			1.13(1.06, 1.20)*
Central			1.17(1.11, 1.24)*
Volta			1.05(0.98, 1.12)
Eastern			1.12(1.06, 1.18)*
Ashanti			1.18(1.12, 1.24)*
Brong Ahafo			1.03(0.98, 1.09)
Northern			1.13(1.06, 1.21)*
Upper East			0.99(0.93, 1.08)
Upper West			1.03(0.94, 1.13)
Wald test (P-value)	133.93(p = 0.000)*	379.88(p = 0.000)*	5.53(p = 0.000)*

Source: GDHS 2003–2014; RR = Risk Ratios; Significance = *<0.05%.

Educational attainment was found to be negatively associated with cumulative fertility with women who had primary and secondary school or higher education having 0.94 and 0.80 times cumulative fertility risk, respectively, compared to those without formal education, after taking into consideration the control factors. Likewise, cumulative fertility was found to be negatively associated with the wealth status of the household. Women from the middle-status households had 0.93 times cumulative fertility risks while women from rich households had 0.76 times cumulative fertility risks compared to their counterparts from poor households. Unemployed women had significantly lower cumulative fertility risk (RR = 0.70) compared to employed women albeit the significance waned considerably after controlling for demographic characteristics and residential factors. Employer status of respondents–whether they are self-employed or employed by someone else–was also associated with cumulative fertility. Self-employed women had at least 1.11 times cumulative fertility risk compared to women who were employed by someone else. However, the regularity or seasonality of women’s employment was not associated with cumulative fertility, after considering the control factors. These two categories of women were found to have virtually the same cumulative fertility.

Additionally, some demographic characteristics have been found to have an association with cumulative fertility as control factors. Expectedly, the current age of women was positively associated with cumulative fertility even after controlling for residential characteristics in model 3. Cumulative fertility risks were 2.67, 5.31, and 7.38 times among women aged 20–29, 30–39, and 40–49, respectively, compared to women aged less than 20. For age at first sex of respondents, the results show a significantly negative association with cumulative fertility. Women who had their first sexual intercourse at ages 20–29 had 0.78 times while those who had theirs at 30 or above had 0.25 times cumulative fertility risks compared to women who had their sexual debut before age 20. Marital status, as may be expected, had a significant association with cumulative fertility as well, with married women (RR = 3.76) and previously married women (RR = 3.53) having considerably higher cumulative fertility risks compared to their counterparts who had never married or lived together with a partner. Religious affiliation was associated with cumulative fertility whereby the risk was 1.12 times among women who identified as traditionalists or spiritualists compared to Christian women. Ethnicity was associated with cumulative fertility, with women of other ethnic groups (Ga-Dangme, RR = 0.91; Ewe, RR = 0.89; Mole-Dagbani, RR = 0.90; Others, RR = 0.92) having significantly lower cumulative fertility than Akan women even though these effects attenuated after adjusting for time and residential factors.

In estimating a temporal association with cumulative fertility, the results indicate that cumulative fertility reduced steadily over time. Cumulative fertility risk was 0.96 and 0.94 times for the years 2008 and 2014, respectively, compared to 2003. For the place of residence, women from rural settings had 1.05 times cumulative fertility risk compared to urban women. At the regional level, the results show some significant disparities in cumulative fertility among the regions. Compared to the Greater Accra region, women residing in the Western (RR = 1.13), Central (RR = 1.17), Eastern (RR = 1.12), and the Ashanti (RR = 1.18), as well as the Northern region (RR = 1.13) region, had significantly higher cumulative fertility.

## Discussion

This study mainly examined the socioeconomic factors associated with cumulative fertility levels in Ghana. The descriptive findings show that while some fluctuations exist in the fertility composition of the study sample, these do not show any considerable changes over the study period. Furthermore, the findings of the multivariate analysis indicate that cumulative fertility is associated with most of the socioeconomic characteristics of women considered in this study. The study links higher cumulative fertility to women without formal education. Bongaarts [30] and Lee [31], among others, have all shown a marked negative relationship between women’s educational attainment and cumulative fertility. Education is expected to lead women into skilled labor force participation, which in turn reduces cumulative fertility [32], and that educated women are more likely to have knowledge about and access to contraception and use contraceptives more effectively [33]. However, highly educated women may also be likely to postpone childbirth because of a considerable part of their reproductive years they may spend in education.

Moreover, cumulative fertility is found to have a significant negative relationship with household wealth status. The findings show pronounced reduced cumulative fertility among women from rich households, disparate from women from poor households who have significantly higher cumulative fertility. The association between wealth and fertility has been fairly debated in the literature. However, this appears to depend on the geographical context or development status of the place under consideration. For instance, studies in developing countries show a negative association between wealth and fertility [23–25], whereas those in developed regions show a positive association with fertility [34]. In this regard, it is empirically unclear why women from poor households in developing countries including Ghana have considerably higher cumulative fertility contrary to their counterparts in the developed world.

The association between labor force participation and cumulative fertility as well appears to be significant, but only when demographic characteristics are not considered. In effect, employed women show significantly higher cumulative fertility than their unemployed counterparts. This finding supports the findings of previous research in sub-Saharan Africa [24]. This is quite understandable since women who are not active in the labor force may likely postpone childbirth until they are fully employed as they may not be able to afford the cost of children. Conversely, Van den Broeck and Maertens [35] in a study in Senegal, and Brewster and Rindfuss [36] in a study in some developed countries show a negative association between female labor force participation and fertility. However, it has been argued that the relationship between fertility and female workforce participation is mediated by country-specific contexts [37,38]. As well, among active women in the labor force, cumulative fertility appears considerably higher among self-employed women than women employed by someone else. Similarly, studies have shown that women with children are more likely to switch to self-employment in developing countries [39–41]. Perhaps, self-employed women in Ghana may have a highly flexible work-life balance that may permit them to easily attain their fertility intentions, unlike women employed by someone else who may be tied to corporate demands who may have to adapt their fertility intentions to accommodate these demands.

Additionally, some demographic factors have shown some significant association with cumulative fertility. The current age of a woman is associated with cumulative fertility. Thus, the cumulative fertility risk increases as women’s age increases [42–44]. This is quite expected as women in Ghana may be expected to have more children as they grow older and expected to have their maximum number of children by the end of their reproductive years. However, it is fair to say that this may also be affected by many different factors including a woman’s age at first birth or marriage among other factors. In a similar vein, the age at sexual debut has a negative relationship with cumulative fertility in Ghana, as cumulative fertility plummeted markedly with the postponement of sexual debut. This has also been evident among both married and never-married women [25]. Understandably, this is maybe because women with delayed sexual debut have shorter active reproductive years than their counterparts who had early sexual debut during their adolescence. Thus, exploring effective ways in which sexual debut can be postponed among young women may be essential to fertility regulation in Ghana.

The association between marriage and cumulative fertility has long been documented as a proximate determinant of fertility by Bongaarts [45] and his colleagues. Marriage is referred to as a proximate determinant of fertility because it is considered the social institution in which childbearing is mainly approved. In this study, marital status is linked to cumulative fertility with women in marital unions and previously married women having significantly higher cumulative fertility than their never-married counterparts. This evidence is expected because, in Ghana, women’s marriage is the only socially and culturally accepted avenue for childbearing, where women who give birth out of wedlock may be stigmatized. The linkage between marriage and fertility as a distal factor has also been observed in the existing literature [24]. It is argued that postponing marriage increases a woman’s likelihood of having low cumulative fertility [43]. However, this may no more be a guarantee amid rising nonmarital fertility levels in the country [46].

Furthermore, this study finds a religious association with cumulative fertility among the study sample. Women who identified with the traditional or spiritual religions are more likely to give birth than Christian women. The association with religious affiliation, and for that matter, the religious disparities in cumulative fertility levels differ substantially across developing countries [47,48]. For instance, in Nepal, fertility was significantly higher among Muslims [23], while in Nigeria, higher fertility was observed among non-Christians [24]. In this study, however, higher fertility levels have been found among traditionalists and spiritualists. This may be the case when religious groups develop strong fertility norms [49], which may include natalism.

Regarding ethnic disparities in fertility, the cumulative fertility level is significantly higher among Akan women than women from all the other ethnic groups in the country, including other miscellaneous ethnic groups. It is, however, unclear why Akan women may have an unexpectedly higher cumulative fertility level. Analogously, considerable ethnic disparities in fertility levels have also been observed in Nigeria [24] and DR Congo [50], all in sub-Saharan Africa. These substantial ethnic differences in fertility levels may be attributable to cultural factors [51].

The study as well shows some temporal reductions in cumulative fertility levels for the study period. However, the pace of reductions in-between the survey periods has not been sharp enough to warrant a substantial decline in cumulative fertility levels over time. This appears to support the widely established evidence that even though cumulative fertility has consistently reduced over the past decades globally, the pace of decline has either slowed or stalled in sub-Saharan African countries [3,4,12]. This trend has been linked to poor socioeconomic development and family planning programs and the desire for higher family sizes [3,8]. The place of residence is also linked to cumulative fertility with rural women significantly having higher cumulative fertility levels than their urban counterparts. This evidence confirms findings from some studies in Africa [24,52] as well as Asia [23] and underscores the need to further understand the rural-urban dynamics of fertility in Ghana and elsewhere across the globe. The study also shows some evidence of considerable regional disparities in cumulative fertility levels in the country. Women in the Western, Central, Eastern, Ashanti, and the Northern region have all shown higher cumulative fertility levels than women in the Greater Accra region which is the most socioeconomically developed region in the country [9]. Thus, cumulative fertility levels are considerably higher in these regions partly due to relatively lower socioeconomic development among women in these areas as well as some unobserved regional contextual factors.

This study, however, has a few potential limitations that should be considered in the interpretation of the findings. First, the study data are based on retrospective information and, therefore, may be subject to recall bias or reporting errors. Second, since the data are cross-sectional, many of the independent variables provided a snapshot of the scenario whereas the outcome variable comprised lifetime fertility which is affected by the lifetime experiences of women. Consequently, there may be potential reverse relationships between the outcome variables and these independent variables. Despite these limitations, the study makes an invaluable contribution to the area of cumulative fertility in Ghana.

## Conclusions

Socioeconomic characteristics–educational attainment, household wealth, employment, and employer status–appear to be significantly associated with cumulative fertility levels in Ghana. Consequently, having no formal education, coming from a poor household, being employed, and self-employed are associated with higher cumulative fertility in Ghana. Similarly, rural residence, early sexual debut, and marriage are significantly linked to higher cumulative fertility. The study provides evidence that even though cumulative fertility has steadily reduced over time, the pace of decline appears to be slow and, therefore, not substantial enough to engender sharp reductions in cumulative fertility levels in the country.

Ultimately, the cumulative fertility of women can be reduced significantly by alleviating socioeconomic inequalities at the individual and household levels. Thus, individual-level policies should prioritize improving women’s educational attainment and household economic wellbeing while placing serious attention on working women as well as self-employed women. Also, comprehensive sexual and reproductive health programs can focus on delaying sexual debut or promoting abstinence among adolescents. Women residing in regions with higher cumulative fertility levels can as well be targeted for specially tailored fertility regulation programs and interventions.

## Supporting information

S1 File (DOCX)
