# Supplementary material for: Socioeconomic determinants of cumulative fertility in Ghana
Source: PLoS One. 2021 Jun 1;16(6):e0252519. doi: 10.1371/journal.pone.0252519 (PMC8168870; doi:10.1371/journal.pone.0252519)
Supplement: S1 File — (DOCX) [file pone.0252519.s001.docx]

**Supplementary file**

# Overdispersion test

summary(fitdispersion <- svyglm(ceb~factor(edu)+factor(wealthstatus)+factor(workstatus)+factor(employer)+factor(empyear), design=des, family=poisson))

## Call:
## svyglm(formula = ceb ~ factor(edu) + factor(wealthstatus) + factor(workstatus) +
## factor(employer) + factor(empyear), design = des, family = poisson)
##
## Survey design:
## svydesign(ids = ~v021, strata = ~v022, weights = ~wght, data = datall[is.na(datall$wght) ==
## F, ], nest = T)
##
## Coefficients:
## Estimate Std. Error t value Pr(>|t|)
## (Intercept) 1.02717 0.02732 37.595 < 2e-16 ***
## factor(edu)2Primary -0.22825 0.02051 -11.131 < 2e-16 ***
## factor(edu)3secodary/higher -0.49771 0.01941 -25.641 < 2e-16 ***
## factor(wealthstatus)2Middle -0.07991 0.02106 -3.795 0.000156 ***
## factor(wealthstatus)3Rich -0.33724 0.01939 -17.396 < 2e-16 ***
## factor(workstatus)2No -0.33826 0.05561 -6.083 1.67e-09 ***
## factor(employer)2Selfemployed 0.61399 0.02484 24.720 < 2e-16 ***
## factor(empyear)2Seasonal -0.07080 0.01828 -3.873 0.000114 ***
## ---
## Signif. codes: 0 '***' 0.001 '**' 0.01 '*' 0.05 '.' 0.1 ' ' 1
##
## (Dispersion parameter for poisson family taken to be 1.809313)
##
## Number of Fisher Scoring iterations: 5

dispersiontest(fitdispersion)

## Overdispersion test
## data: fitdispersion
## z = 36.866, p-value < 2.2e-16
## alternative hypothesis: true dispersion is greater than 1
## sample estimates:
## dispersion
## 1.807951

# Survey negative binomial models

**# Model 1**

fit1<-svyglm.nb(ceb~factor(edu)+factor(wealthstatus)+factor(workstatus)+factor(employer)+factor(empyear), design=des)
# Model summary
summary(fit1)

## Survey-sampled mle:
## survey::svymle(loglike = sjstats_loglik, gradient = sjstats_score,
## design = design, formulas = list(theta = ~1, eta = formula),
## start = c(mod$theta, stats::coef(mod)), na.action = "na.omit")
## Coef SE p.value
## theta.(Intercept) 3.41691752 0.12793092 <0.001
## eta.(Intercept) 1.02545207 0.02715682 <0.001
## eta.factor(edu)2Primary -0.25065462 0.02122795 <0.001
## eta.factor(edu)3secodary/higher -0.52333249 0.01987969 <0.001
## eta.factor(wealthstatus)2Middle -0.08746624 0.02187181 <0.001
## eta.factor(wealthstatus)3Rich -0.34410436 0.02004418 <0.001
## eta.factor(workstatus)2No -0.35110924 0.05688462 <0.001
## eta.factor(employer)2Selfemployed 0.64628856 0.02525496 <0.001
## eta.factor(empyear)2Seasonal -0.08017439 0.01986639 <0.001
## Stratified 1 - level Cluster Sampling design (with replacement)
## With (1225) clusters.
## stats::update(design, scaled.weights = dw/mean(dw, na.rm = TRUE))

#Risk ratios and CIs

exp(coef(fit1))

## theta.(Intercept) eta.(Intercept)
## 30.4753306 2.7883557
## eta.factor(edu)2Primary eta.factor(edu)3secodary/higher
## 0.7782911 0.5925426
## eta.factor(wealthstatus)2Middle eta.factor(wealthstatus)3Rich
## 0.9162498 0.7088549
## eta.factor(workstatus)2No eta.factor(employer)2Selfemployed
## 0.7039069 1.9084446
## eta.factor(empyear)2Seasonal
## 0.9229554

exp(confint(fit1))

## 2.5 % 97.5 %
## theta.(Intercept) 23.7166546 39.1600667
## eta.(Intercept) 2.6438222 2.9407906
## eta.factor(edu)2Primary 0.7465739 0.8113558
## eta.factor(edu)3secodary/higher 0.5698991 0.6160858
## eta.factor(wealthstatus)2Middle 0.8778020 0.9563816
## eta.factor(wealthstatus)3Rich 0.6815469 0.7372572
## eta.factor(workstatus)2No 0.6296438 0.7869289
## eta.factor(employer)2Selfemployed 1.8162787 2.0052874
## eta.factor(empyear)2Seasonal 0.8877085 0.9596017

**# Model 2**

fit2<-svyglm.nb(ceb~factor(edu)+factor(wealthstatus)+factor(workstatus)+factor(employer)+factor(empyear)+factor(agegroup)+factor(ageatsex)+factor(maristatus)+factor(ethnicity)+factor(religion), design=des)

# Model summary
summary(fit2)

## Survey-sampled mle:
## survey::svymle(loglike = sjstats_loglik, gradient = sjstats_score,
## design = design, formulas = list(theta = ~1, eta = formula),
## start = c(mod$theta, stats::coef(mod)), na.action = "na.omit")
## Coef SE
## theta.(Intercept) 1.726264e+04 1.162775e+03
## eta.(Intercept) -1.421728e+00 8.372930e-02
## eta.factor(edu)2Primary -6.486775e-02 1.820413e-02
## eta.factor(edu)3secodary/higher -2.363220e-01 1.770281e-02
## eta.factor(wealthstatus)2Middle -8.724037e-02 1.802029e-02
## eta.factor(wealthstatus)3Rich -3.196727e-01 1.642822e-02
## eta.factor(workstatus)2No -7.218344e-02 4.374844e-02
## eta.factor(employer)2Selfemployed 1.107055e-01 1.823203e-02
## eta.factor(empyear)2Seasonal 1.461800e-02 1.690444e-02
## eta.factor(agegroup)20-29 9.768429e-01 7.040680e-02
## eta.factor(agegroup)30-39 1.658271e+00 7.132694e-02
## eta.factor(agegroup)40-49 1.988136e+00 7.241104e-02
## eta.factor(ageatsex)20-29 -2.569703e-01 1.781030e-02
## eta.factor(ageatsex)30+ -1.400345e+00 2.409479e-01
## eta.factor(maristatus)2Married/livingwithpartner 1.336577e+00 5.184558e-02
## eta.factor(maristatus)3Separated/divorced/widowed1.161673e+00 5.363329e-02
## eta.factor(ethnicity)2Ga/Dangme -8.922112e-02 2.629215e-02
## eta.factor(ethnicity)3Ewe -1.154806e-01 1.981685e-02
## eta.factor(ethnicity)4Mole-dagbani -1.008193e-01 2.258829e-02
## eta.factor(ethnicity)5Other -8.252803e-02 2.523561e-02
## eta.factor(religion)2Islam 1.895409e-02 2.415728e-02
## eta.factor(religion)3Traditional/Spiritualist 9.742806e-02 3.211298e-02
## eta.factor(religion)Other 1.853349e-02 3.693706e-02
## p.value
## theta.(Intercept) < 0.001
## eta.(Intercept) < 0.001
## eta.factor(edu)2Primary < 0.001
## eta.factor(edu)3secodary/higher < 0.001
## eta.factor(wealthstatus)2Middle < 0.001
## eta.factor(wealthstatus)3Rich < 0.001
## eta.factor(workstatus)2No 0.09895
## eta.factor(employer)2Selfemployed < 0.001
## eta.factor(empyear)2Seasonal 0.38718
## eta.factor(agegroup)20-29 < 0.001
## eta.factor(agegroup)30-39 < 0.001
## eta.factor(agegroup)40-49 < 0.001
## eta.factor(ageatsex)20-29 < 0.001
## eta.factor(ageatsex)30+ < 0.001
## eta.factor(maristatus)2Married/livingwithpartner < 0.001
## eta.factor(maristatus)3Separated/divorced/widowed < 0.001
## eta.factor(ethnicity)2Ga/Dangme < 0.001
## eta.factor(ethnicity)3Ewe < 0.001
## eta.factor(ethnicity)4Mole-dagbani < 0.001
## eta.factor(ethnicity)5Other 0.00107
## eta.factor(religion)2Islam 0.43268
## eta.factor(religion)3Traditional/Spiritualist 0.00241
## eta.factor(religion)Other 0.61584
## Stratified 1 - level Cluster Sampling design (with replacement)
## With (1218) clusters.
## stats::update(design, scaled.weights = dw/mean(dw, na.rm = TRUE))

#Risk ratios and CI

exp(coef(fit2))

## theta.(Intercept)
## Inf
## eta.(Intercept)
## 0.2412968
## eta.factor(edu)2Primary
## 0.9371914
## eta.factor(edu)3secodary/higher
## 0.7895264
## eta.factor(wealthstatus)2Middle
## 0.9164568
## eta.factor(wealthstatus)3Rich
## 0.7263868
## eta.factor(workstatus)2No
## 0.9303602
## eta.factor(employer)2Selfemployed
## 1.1170659
## eta.factor(empyear)2Seasonal
## 1.0147254
## eta.factor(agegroup)20-29
## 2.6560576
## eta.factor(agegroup)30-39
## 5.2502244
## eta.factor(agegroup)40-49
## 7.3019118
## eta.factor(ageatsex)20-29
## 0.7733912
## eta.factor(ageatsex)30+
## 0.2465120
## eta.factor(maristatus)2Married/livingwithpartner
## 3.8059923
## eta.factor(maristatus)3Separated/divorced/widowed
## 3.1952739
## eta.factor(ethnicity)2Ga/Dangme
## 0.9146433
## eta.factor(ethnicity)3Ewe
## 0.8909379
## eta.factor(ethnicity)4Mole-dagbani
## 0.9040964
## eta.factor(ethnicity)5Other
## 0.9207856
## eta.factor(religion)2Islam
## 1.0191349
## eta.factor(religion)3Traditional/Spiritualist
## 1.1023321
## eta.factor(religion)Other
## 1.0187063

exp(confint(fit2))

## 2.5 % 97.5 %
## theta.(Intercept) Inf Inf
## eta.(Intercept) 0.2047769 0.2843296
## eta.factor(edu)2Primary 0.9043424 0.9712336
## eta.factor(edu)3secodary/higher 0.7626021 0.8174013
## eta.factor(wealthstatus)2Middle 0.8846533 0.9494036
## eta.factor(wealthstatus)3Rich 0.7033706 0.7501561
## eta.factor(workstatus)2No 0.8539106 1.0136543
## eta.factor(employer)2Selfemployed 1.0778533 1.1577050
## eta.factor(empyear)2Seasonal 0.9816562 1.0489085
## eta.factor(agegroup)20-29 2.3137003 3.0490733
## eta.factor(agegroup)30-39 4.5652468 6.0379772
## eta.factor(agegroup)40-49 6.3357815 8.4153652
## eta.factor(ageatsex)20-29 0.7468598 0.8008651
## eta.factor(ageatsex)30+ 0.1537244 0.3953058
## eta.factor(maristatus)2Married/livingwithpartner 3.4382454 4.2130726
## eta.factor(maristatus)3Separated/divorced/widowed 2.8764402 3.5494481
## eta.factor(ethnicity)2Ga/Dangme 0.8687040 0.9630120
## eta.factor(ethnicity)3Ewe 0.8569970 0.9262230
## eta.factor(ethnicity)4Mole-dagbani 0.8649431 0.9450220
## eta.factor(ethnicity)5Other 0.8763507 0.9674736
## eta.factor(religion)2Islam 0.9720060 1.0685488
## eta.factor(religion)3Traditional/Spiritualist 1.0350894 1.1739432
## eta.factor(religion)Other 0.9475630 1.0951910

**# Model 3**

fit3<-svyglm.nb(ceb~factor(edu)+factor(wealthstatus)+factor(workstatus)+factor(employer)+factor(empyear)+factor(agegroup)+factor(ageatsex)+factor(maristatus)+factor(ethnicity)+factor(religion)+factor(year)+factor(residence)+factor(region), design=des)

# Model summary
summary(fit3)

## Survey-sampled mle:
## survey::svymle(loglike = sjstats_loglik, gradient = sjstats_score,
## design = design, formulas = list(theta = ~1, eta = formula),
## start = c(mod$theta, stats::coef(mod)), na.action = "na.omit")
## Coef SE
## theta.(Intercept) 1.821992e+04 1.037665e+03
## eta.(Intercept) -1.555787e+00 8.667474e-02
## eta.factor(edu)2Primary -5.949043e-02 1.853931e-02
## eta.factor(edu)3secodary/higher -2.238485e-01 1.779686e-02
## eta.factor(wealthstatus)2Middle -7.585103e-02 1.856136e-02
## eta.factor(wealthstatus)3Rich -2.679511e-01 2.263019e-02
## eta.factor(workstatus)2No -7.343226e-02 4.365024e-02
## eta.factor(employer)2Selfemployed 1.045585e-01 1.804790e-02
## eta.factor(empyear)2Seasonal 4.041998e-03 1.731956e-02
## eta.factor(agegroup)20-29 9.820778e-01 7.122383e-02
## eta.factor(agegroup)30-39 1.669128e+00 7.210592e-02
## eta.factor(agegroup)40-49 1.998966e+00 7.329765e-02
## eta.factor(ageatsex)20-29 -2.531318e-01 1.773439e-02
## eta.factor(ageatsex)30+ -1.401349e+00 2.400402e-01
## eta.factor(maristatus)2Married/livingwithpartner 1.323411e+00 5.200611e-02
## eta.factor(maristatus)3Separated/divorced/widowed1.155985e+00 5.350878e-02
## eta.factor(ethnicity)2Ga/Dangme -2.482913e-02 2.816511e-02
## eta.factor(ethnicity)3Ewe -5.535656e-02 2.675240e-02
## eta.factor(ethnicity)4Mole-dagbani -4.763671e-02 2.732036e-02
## eta.factor(ethnicity)5Other -4.183339e-02 2.464124e-02
## eta.factor(religion)2Islam 3.197045e-02 2.413139e-02
## eta.factor(religion)3Traditional/Spiritualist 1.173662e-01 3.235821e-02
## eta.factor(religion)Other 4.653254e-02 3.770086e-02
## eta.factor(year)2008 -3.939295e-02 1.640224e-02
## eta.factor(year)2014 -5.760163e-02 1.533212e-02
## eta.factor(residence)2Rural 4.968764e-02 1.882414e-02
## eta.factor(region)2Western 1.200186e-01 3.115803e-02
## eta.factor(region)3Central 1.579275e-01 2.936311e-02
## eta.factor(region)4Volta 4.584409e-02 3.523617e-02
## eta.factor(region)5Eastern 1.119924e-01 2.649657e-02
## eta.factor(region)6Ashanti 1.640882e-01 2.413842e-02
## eta.factor(region)7Brong 3.149771e-02 2.893949e-02
## eta.factor(region)8Northern 1.224507e-01 3.472155e-02
## eta.factor(region)91UWest 3.300710e-02 4.749642e-02
## eta.factor(region)9UEast -1.717684e-03 3.876453e-02
## p.value
## theta.(Intercept) < 0.001
## eta.(Intercept) < 0.001
## eta.factor(edu)2Primary 0.00133
## eta.factor(edu)3secodary/higher < 0.001
## eta.factor(wealthstatus)2Middle < 0.001
## eta.factor(wealthstatus)3Rich < 0.001
## eta.factor(workstatus)2No 0.09251
## eta.factor(employer)2Selfemployed < 0.001
## eta.factor(empyear)2Seasonal 0.81547
## eta.factor(agegroup)20-29 < 0.001
## eta.factor(agegroup)30-39 < 0.001
## eta.factor(agegroup)40-49 < 0.001
## eta.factor(ageatsex)20-29 < 0.001
## eta.factor(ageatsex)30+ < 0.001
## eta.factor(maristatus)2Married/livingwithpartner < 0.001
## eta.factor(maristatus)3Separated/divorced/widowed < 0.001
## eta.factor(ethnicity)2Ga/Dangme 0.37802
## eta.factor(ethnicity)3Ewe 0.03853
## eta.factor(ethnicity)4Mole-dagbani 0.08122
## eta.factor(ethnicity)5Other 0.08956
## eta.factor(religion)2Islam 0.18522
## eta.factor(religion)3Traditional/Spiritualist < 0.001
## eta.factor(religion)Other 0.21711
## eta.factor(year)2008 0.01632
## eta.factor(year)2014 < 0.001
## eta.factor(residence)2Rural 0.00830
## eta.factor(region)2Western < 0.001
## eta.factor(region)3Central < 0.001
## eta.factor(region)4Volta 0.19324
## eta.factor(region)5Eastern < 0.001
## eta.factor(region)6Ashanti < 0.001
## eta.factor(region)7Brong 0.27642
## eta.factor(region)8Northern < 0.001
## eta.factor(region)91UWest 0.48709
## eta.factor(region)9UEast 0.96466
## Stratified 1 - level Cluster Sampling design (with replacement)
## With (1218) clusters.
## stats::update(design, scaled.weights = dw/mean(dw, na.rm = TRUE))

#Risk ratios and CI

exp(coef(fit3))

## theta.(Intercept)
## Inf
## eta.(Intercept)
## 0.2110233
## eta.factor(edu)2Primary
## 0.9422446
## eta.factor(edu)3secodary/higher
## 0.7994362
## eta.factor(wealthstatus)2Middle
## 0.9269543
## eta.factor(wealthstatus)3Rich
## 0.7649451
## eta.factor(workstatus)2No
## 0.9291991
## eta.factor(employer)2Selfemployed
## 1.1102203
## eta.factor(empyear)2Seasonal
## 1.0040502
## eta.factor(agegroup)20-29
## 2.6699983
## eta.factor(agegroup)30-39
## 5.3075371
## eta.factor(agegroup)40-49
## 7.3814224
## eta.factor(ageatsex)20-29
## 0.7763656
## eta.factor(ageatsex)30+
## 0.2462646
## eta.factor(maristatus)2Married/livingwithpartner
## 3.7562114
## eta.factor(maristatus)3Separated/divorced/widowed
## 3.1771529
## eta.factor(ethnicity)2Ga/Dangme
## 0.9754766
## eta.factor(ethnicity)3Ewe
## 0.9461477
## eta.factor(ethnicity)4Mole-dagbani
## 0.9534801
## eta.factor(ethnicity)5Other
## 0.9590296
## eta.factor(religion)2Islam
## 1.0324870
## eta.factor(religion)3Traditional/Spiritualist
## 1.1245312
## eta.factor(religion)Other
## 1.0476322
## eta.factor(year)2008
## 0.9613729
## eta.factor(year)2014
## 0.9440259
## eta.factor(residence)2Rural
## 1.0509428
## eta.factor(region)2Western
## 1.1275178
## eta.factor(region)3Central
## 1.1710812
## eta.factor(region)4Volta
## 1.0469112
## eta.factor(region)5Eastern
## 1.1185044
## eta.factor(region)6Ashanti
## 1.1783182
## eta.factor(region)7Brong
## 1.0319990
## eta.factor(region)8Northern
## 1.1302633
## eta.factor(region)91UWest
## 1.0335579
## eta.factor(region)9UEast
## 0.9982838

exp(confint(fit3))

## 2.5 % 97.5 %
## theta.(Intercept) Inf Inf
## eta.(Intercept) 0.1780544 0.2500968
## eta.factor(edu)2Primary 0.9086214 0.9771120
## eta.factor(edu)3secodary/higher 0.7720316 0.8278135
## eta.factor(wealthstatus)2Middle 0.8938381 0.9612974
## eta.factor(wealthstatus)3Rich 0.7317579 0.7996375
## eta.factor(workstatus)2No 0.8530090 1.0121944
## eta.factor(employer)2Selfemployed 1.0716347 1.1501952
## eta.factor(empyear)2Seasonal 0.9705390 1.0387185
## eta.factor(agegroup)20-29 2.3221226 3.0699890
## eta.factor(agegroup)30-39 4.6080414 6.1132156
## eta.factor(agegroup)40-49 6.3936519 8.5217959
## eta.factor(ageatsex)20-29 0.7498437 0.8038256
## eta.factor(ageatsex)30+ 0.1538436 0.3942072
## eta.factor(maristatus)2Married/livingwithpartner 3.3922070 4.1592757
## eta.factor(maristatus)3Separated/divorced/widowed 2.8608254 3.5284573
## eta.factor(ethnicity)2Ga/Dangme 0.9230871 1.0308394
## eta.factor(ethnicity)3Ewe 0.8978159 0.9970814
## eta.factor(ethnicity)4Mole-dagbani 0.9037671 1.0059277
## eta.factor(ethnicity)5Other 0.9138130 1.0064835
## eta.factor(religion)2Islam 0.9847906 1.0824934
## eta.factor(religion)3Traditional/Spiritualist 1.0554269 1.1981601
## eta.factor(religion)Other 0.9730111 1.1279760
## eta.factor(year)2008 0.9309583 0.9927810
## eta.factor(year)2014 0.9160796 0.9728248
## eta.factor(residence)2Rural 1.0128752 1.0904411
## eta.factor(region)2Western 1.0607222 1.1985197
## eta.factor(region)3Central 1.1055874 1.2404548
## eta.factor(region)4Volta 0.9770499 1.1217677
## eta.factor(region)5Eastern 1.0619004 1.1781257
## eta.factor(region)6Ashanti 1.1238696 1.2354047
## eta.factor(region)7Brong 0.9750928 1.0922263
## eta.factor(region)8Northern 1.0559044 1.2098588
## eta.factor(region)91UWest 0.9416853 1.1343938
## eta.factor(region)9UEast 0.9252468 1.0770862

# Model fit test for models 1-3

**# Model 1**
regTermTest(fit3, test.terms= ~factor(edu)+factor(wealthstatus)+factor(workstatus)+factor(employer)+factor(empyear), method="Wald", df=NULL)

## Wald test for factor(edu) factor(wealthstatus) factor(workstatus) factor(employer) factor(empyear)
## in survey::svymle(loglike = sjstats_loglik, gradient = sjstats_score,
## design = design, formulas = list(theta = ~1, eta = formula),
## start = c(mod$theta, stats::coef(mod)), na.action = "na.omit")
## F = 133.9326 on 7 and 10471 df: p= < 2.22e-16

**# Model 2**

regTermTest(fit2, test.terms = ~factor(agegroup)+factor(ageatsex)+factor(maristatus)+factor(ethnicity)+factor(religion), method="Wald",df =NULL)

## Wald test for factor(agegroup) factor(ageatsex) factor(maristatus) factor(ethnicity) factor(religion)
## in survey::svymle(loglike = sjstats_loglik, gradient = sjstats_score,
## design = design, formulas = list(theta = ~1, eta = formula),
## start = c(mod$theta, stats::coef(mod)), na.action = "na.omit")
## F = 379.8843 on 12 and 10483 df: p= < 2.22e-16

**# Model 3**

regTermTest(fit3, test.terms = ~factor(year)+factor(residence)+factor(region), method="Wald",df =NULL)

## Wald test for factor(year) factor(residence) factor(region)
## in survey::svymle(loglike = sjstats_loglik, gradient = sjstats_score,
## design = design, formulas = list(theta = ~1, eta = formula),
## start = c(mod$theta, stats::coef(mod)), na.action = "na.omit")
## F = 5.536756 on 6 and 10471 df: p= 9.7064e-06
